# Song convergence in multiple urban populations of silvereyes (*Zosterops lateralis*)

**DOI:** 10.1002/ece3.320

**Published:** 2012-07-16

**Authors:** Dominique A Potvin, Kirsten M Parris

**Affiliations:** 1 Department of Zoology, University of MelbourneVIC, 3010, Australia; 2 School of Botany, University of MelbourneVIC, 3010, Australia

**Keywords:** Acoustic adaptation, cultural evolution, silvereyes, song dialects, urban noise, *Zosterops lateralis*

## Abstract

Recent studies have revealed differences between urban and rural vocalizations of numerous bird species. These differences include frequency shifts, amplitude shifts, altered song speed, and selective meme use. If particular memes sung by urban populations are adapted to the urban soundscape, “urban-typical” calls, memes, or repertoires should be consistently used in multiple urban populations of the same species, regardless of geographic location. We tested whether songs or contact calls of silvereyes (*Zosterops lateralis*) might be subject to such convergent cultural evolution by comparing syllable repertoires of geographically dispersed urban and rural population pairs throughout southeastern Australia. Despite frequency and tempo differences between urban and rural calls, call repertoires were similar between habitat types. However, certain song syllables were used more frequently by birds from urban than rural populations. Partial redundancy analysis revealed that both geographic location and habitat characteristics were important predictors of syllable repertoire composition. These findings suggest convergent cultural evolution: urban populations modify both song and call syllables from their local repertoire in response to noise.

## Introduction

The acoustic adaptation hypothesis (Morton 1975) proposes that animals adjust their acoustic signals according to their environment to increase transmission and reduce degradation of the signal. Recently, studies testing this hypothesis in urban environments have shown that urban birds adjust their songs in noisy conditions (Slabbekoorn and Peet 2003). For example, many species increase the minimum frequency of songs in urban areas, a modification that may be an adaptive response to minimize interference from low-frequency masking noise (Slabbekoorn and Peet 2003; Brumm 2006; Potvin et al. 2011). However, the adaptive value of these frequency shifts has recently been debated. Frequency modifications appear to produce minor increases in transmission distance (Nemeth and Brumm 2010), and thus, frequency shifts may simply be a consequence of singing at higher amplitudes – another common vocal adjustment to environmental noise known as the Lombard effect (Nemeth and Brumm 2010). Although this would explain frequency shifts in apparently innate vocalizations (Potvin et al. 2011), there is now evidence that birds are able to adjust amplitude and frequency independently under noisy conditions (Cardoso and Atwell 2011a), restoring support for the AAH.

In addition to frequency shifts, other modifications that may reduce song degradation in a given habitat include altering tempo or using specific syllables or songs that transmit well through particular acoustic environments (Mockford et al. 2011). Examples of this behavioral practice are found in multiple species, including the great tit *Parus major* and the satin bowerbird *Ptilonorhynchus violaceus*. Individuals living in densely wooded habitats produce fewer notes per phrase and fewer frequency modulations than those living in open habitats (Hunter and Krebs 1979; Nicholls and Goldizen 2006). While these examples involve acoustic properties of natural rather than urban surroundings, one might expect analogous responses by populations of the same species inhabiting both urban and rural habitats.

Although the acoustic environment plays a part in the song structure of many species, its influence may be constrained by the process of cultural transmission. Passerines often learn songs or syllables from other individuals in their population, leading to population-specific repertoires or “dialects”. Where populations are separated by large geographic distances or barriers that prevent dispersal, these dialects frequently become geographically distinct. Dialects are therefore commonly defined by geographic location, with adjacent populations singing similar songs and populations that are separated by large distances singing very different songs due to the processes of cultural transmission of song, dispersal, and learning (Baker and Cunningham 1985).

To date, few studies have investigated the potential for different dialects to develop in urban areas. Evidence for cultural evolution, or the development of a distinct urban repertoire, has only recently been found in dark-eyed juncos *Junco hyemalis* (Cardoso and Atwell 2011a). In this species, dialects in urban areas have developed over time to include memes and other spectral characteristics specific to urban populations. However, as this comparison involved only one urban and one rural population, we still cannot be sure that the syllables included in these urban repertoires are indeed adapted to urban-specific acoustic features, rather than a by-product of low dispersal between the two specific urban and rural populations used in the study (cultural drift). Although there is evidence that individuals that sing differently under such circumstances are probably able to disperse between adjacent urban and rural habitats (Mockford and Marshall 2009), convincing evidence of urban acoustic adaptation rather than natural patterns of random cultural drift would ideally require analysis of repertoires in paired rural–urban sites across large geographic scales.

**Figure 1:**
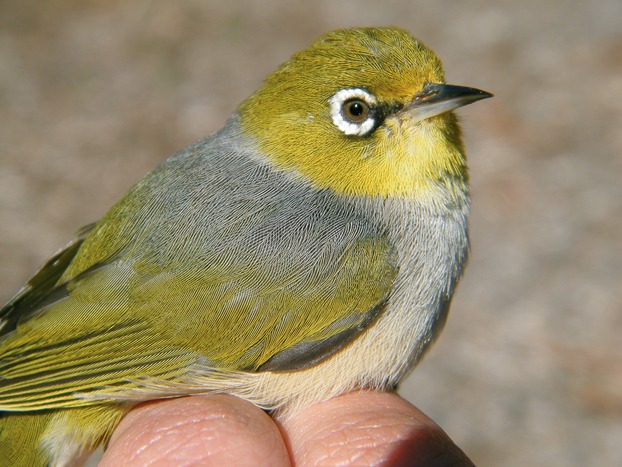
Photograph of a silvereye *Zosterops lateralis* from Canberra, Australia.

Here, we report on such an investigation of syllable repertoires in songs and contact calls of silvereyes (*Zosterops lateralis:* Fig. 1) across seven pairs of urban and rural populations, distributed over an area of 1 million square kilometers of southeastern Australia. The silvereye is an Australian passerine whose song and call frequencies are higher in urban areas (Potvin et al. 2011). Our objective in this study was to test whether urban habitats were consistently selecting for potentially adaptive syllable repertoires. To do this, we considered measurable differences in the syllable repertoires of urban and rural populations of silvereyes, and whether any such differences could be attributed to habitat-specific features such as noise.

We analyzed spectral features of both song and call syllables to test the hypothesis that acoustic adaptation is promoting converging cultural evolution of repertoires in multiple urban populations. First, we analyzed syllable frequency range, duration, and tempo, with the prediction that urban habitat would likely select for narrower, longer, and slower/simpler syllables, as these syllables may be more detectible and distinguishable in noisy urban areas (Brumm et al. 2004; Patricelli and Blickley 2006; Derryberry 2009). For these reasons, we also predicted that urban populations might use fewer trills in song repertoires than rural populations, since trills are by nature fast syllables. To determine whether urban habitats selected for overall similar population repertoires, we also analyzed the predictive effects of habitat type versus geographic location on the similarity of population song syllable repertoires (presence and abundance of syllable types). If urbanization is promoting convergent evolution of repertoires, we would expect habitat features to be highly predictive of repertoire or dialect similarity, comparable to the potential predictive effects of geographic location.

## Methods

### Species

The silvereye is a native Australian passerine common in both urban and rural environments. Silvereyes sing songs and contact calls with a higher minimum frequency in urban than rural environments, and urban songs are also slower (contain fewer syllables/sec: Potvin et al. 2011). Males of this species have a large repertoire consisting of syllables that are arranged and rearranged in series to form unique songs, which include between four and 20 syllables in seemingly random order. The inclusion and order of syllables is inconsistent between songs (D. Potvin, pers. obs.). Silvereyes also possess four common types of contact calls – linear call, short call, variable call, and chip call (Appendix S1) – and all individuals may use all types of calls (Bruce and Kikkawa 1988). The purpose of each call is unknown, but calls are thought to contain identifying information about individuals (Robertson 1996).

### Field locations

Study sites were paired urban and rural locations in distinct geographic areas around Australia, as follows: Melbourne, Victoria (−37.5, 144.5; Darebin Parklands and Lerderderg State Park); Adelaide, South Australia (−35.0, 138.5; Glenalta and Coorong National Park); Sydney, New South Wales (−34.0, 151.0; Poulton Park and Munghorn Gap Nature Reserve); Grafton, New South Wales (−30.0, 153.0; Susan Island and Lamington National Park); Brisbane, Queensland (−27.5, 153.0; Kingfisher Park and Mount Coot-Tha State Forest); Hobart, Tasmania (−43.0, 147.5; Seven Mile Beach/Hobart Airport and Mount Wellington Reserve); Canberra, A.C.T. (−35.0, 149; Australian National Botanic Gardens and Namadgi National Park). All these sites have breeding, resident populations of silvereyes (see Appendix S2 for map).

### Field methods

We performed fieldwork in the summer to ensure sampling of resident, breeding populations of silvereyes. Between September 2009 and February 2010, we caught silvereyes in mistnets over the course of 2–8 days at each site. We fitted each captured individual with an ABBBS (Australian Bird and Bat Banding Scheme) aluminum numbered band, as well as three color bands. During subsequent days, we recorded songs and calls of between four and nine banded individuals with Marantz Professional PMD660 Solid State recorders (Marantz, Kanagawa, Japan) and Sennheiser ME67 directional microphones (Sennheiser, Hanover, Germany) at a sampling rate of 48 kHz. A total of 81 complete dawn choruses (songs) were recorded, along with any contact calling (opportunistically recorded once dawn chorus finished) between dawn and 12:00 pm at every site.

We took sound level readings at each site using 10 separate locations, each 20 m apart. We took a 1-min reading at each location at 6:00, 9:00, and 12:00 hour using a Lutron SL-4001 Sound Level Meter (Lutron Electronics Inc., Coopersburg, Pennsylvania) using a slow response measurement with “A” weighting. We then calculated average levels of background noise for each study site.

### Sound analysis

To analyze the recorded vocalizations, we first generated spectrograms of all recordings using the program RavenPro 1.4 (Bioacoustics Research Program, 2011). We identified silvereye vocalizations both visually and aurally using shape, energy, and timbre. A syllable was defined as one or more notes that always occurred together (Stewart and Macdougall-Shackleton 2008). For each syllable, we determined frequency range (maximum–minimum frequency present in the syllable), duration of the syllable in seconds, and the number of “peaks” in the syllable. Peaks were used as a measure of syllable complexity (peaks/sec), as they indicate frequency modulation: peaks were defined as the highest points above the average frequency, to which frequency was modulated for each song and call syllable (see Appendix S1). We then divided each song syllable into three equal temporal sections and used the number of peaks, relative energy, and overall shape in each section to categorize the syllable as objectively as possible, which resulted in 198 syllable types over the whole study. To confirm syllable categorization, we repeated the analysis and categorization for two populations and compared results. Out of 1006 syllables categorized, 8 (0.8%) were misplaced, resulting in 99.2% accuracy in reliability of classification.

Trills were a noticeably different syllable type from all others, consisting of very fast, repeated notes rather than a single modulating sound. We therefore also included a count of the number of trills included in each song. In contrast to song syllables, call syllables were stereotypical and were classified as either a linear call, short call, variable call, chip call, or other (with “other” constituting around 2% of all calls; Appendix S1). Percentage use of each call syllable type was calculated for each population. We performed all spectral analyses blind to the identity of the bird and the site.

To standardize syllable repertoire size per population, we took the total number of different syllables in the recordings from a given population and divided it by the total number of all syllables recorded in that population to come up with a repertoire size score. As the likelihood of detecting new syllables increases with the number of syllables recorded, populations that have larger repertoires will have a higher repertoire size score, regardless of the number of syllables recorded.

### Statistical analysis

We used Bayesian linear regression modeling in OpenBUGS (Spiegelhalter et al. 2006; McCarthy 2007) to assess the effect of habitat (rural or urban) on each of seven response variables: syllable repertoire size (songs only), syllables per song, percentage of trill syllables in repertoire, percentage call-type use (calls only), the frequency range of the syllable, syllable duration (s), and syllable peak rate (tempo). We included uninformative priors in the models to reflect a lack of prior information. We also included a random site effect and a random “pair” effect to account for variation in song due to the geographic location of each pair of sites. This analysis contained no prior information and therefore is equivalent to a general linear mixed model (GLMM) with two random effects. We discarded the first 100,000 samples as a burn-in and checked for convergence. We estimated the mean, standard deviation (SD), and 95% credible interval of model coefficients using 200,000 samples drawn from the posterior distribution, and report these values (means and 95% credible intervals) in the results. We interpreted the importance of the effect of habitat on vocal signals using these output variables, as described in Cumming and Finch (2005). The 95% credible intervals are equivalent to the 95% confidence intervals that would be calculated in an analogous frequentist analysis of the standard error (SE) of the mean (McCarthy 2007).

To analyze the similarity of each population's song syllable repertoire to those of other populations, we used the program CANOCO 4.0 (Ter Braak and Milauer 1998). We used partial redundancy analyses (RDA) in CANOCO to evaluate how much variation in syllable repertoire could be explained by habitat type and spatial position (geographic location). Redundancy analyses are analogous to multivariate regressions, determining the effect of one set of variables on the variance of another set, using derived values. All syllables present, and the relative abundance of each in each population repertoire were included in a matrix and then transformed to give the Hellinger distance between populations, which is suitable for abundance data that contain many zeros (Rao 1995; Legendre and Legendre 1998). We then constructed a second matrix with three habitat-based variables: open/closed habitat type, urban/rural habitat type, and background noise level. The third matrix (spatial matrix) contained the normalized geographic co-ordinates of the field sites in decimal degrees. Four steps were then followed to determine the variance explained by each set of variables: an RDA of the syllable matrix constrained by the habitat matrix; an RDA of the syllable matrix constrained by the spatial matrix; a partial RDA of the syllable matrix constrained by the habitat matrix, after accounting for variance explained by the spatial matrix; and vice versa. We used forward selection of explanatory variables (similar to forward stepwise regression).

### Ethical note

Procedures were undertaken with the approval of the following agencies: Animal Ethics Committee at the University of Melbourne, Director-General's Animal Care and Ethics Committee at the NSW Department of Primary Industries, and Wildlife Ethics Committee at the SA Department for Environment and Heritage. All mist-netting and banding was conducted under national Australian Bird and Bat Banding Scheme licenses 2888 and 1405.

## Results

### Song syllables

**Figure 2:**
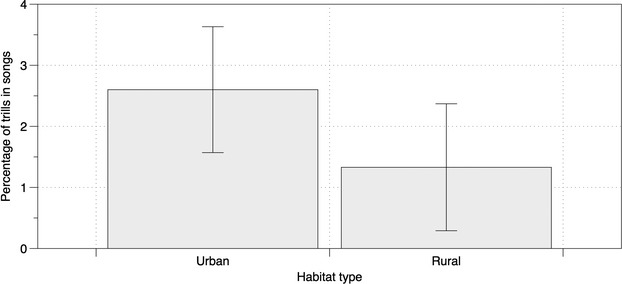
The percentage of all syllable types classified as trills within all urban and rural songs across the study. Error bars represent standard deviation.

Regression modeling revealed that urban populations and rural populations had a similar diversity of unique song syllables in their repertoire (mean overall repertoire size score difference = 1.879; 95% CI = −6.082, 9.685; descriptive statistics in Table 1). Urban songs contained an average of 4.0 fewer syllables per song than rural songs (95% CI = −9.951, 1.833), translating to urban songs having up to 27% fewer syllables than those sung in rural areas (Table 1). Within their songs, urban birds used a higher percentage of trill syllable types than rural birds (mean difference = 1.13%; 95% CI = −1.052, 3.196, Fig. 2), and urban syllables had a larger frequency range by about 206 Hz (95% CI = 117.2, 352.9). This represents an approximate increase of 11% in the frequency range of urban syllables, compared with the range of rural syllables (Table 1). Urban and rural syllables were similar in length; however, the tempo (peaks/sec) of urban syllables was slightly slower than those in rural areas (mean difference = −1.215 s; 95% CI = −4.319, 1.917).

**Table 1:** Descriptive statistics for song syllable and call variables by habitat type. Data are means ± standard deviation

	RSS	Syllables per song	% Trills in rep	Frequency range (Hz)	Duration (s)	Tempo (peaks/sec)	% Linear calls	% Short calls	% Variable calls	Frequency range (Hz)	Duration (s)	Tempo (peaks/sec)
Rural songs/calls	19.39 ± 9.90	14.75 ± 5.71	2.12 ± 3.17	2140.11 ± 492.39	0.13 ± 0.03	17.67 ± 3.86	19.90 ± 11.25	51.60 ± 21.93	24.56 ± 18.26	2300.70 ± 254.02	0.19 ± 0.05	13.59 ± 2.74
Urban songs/calls	20.78 ± 7.28	10.74 ± 3.48	15.23 ± 22.63	2346.15 ± 302.63	0.14 ± 0.03	16.11 ± 4.76	20.17 ± 15.11	39.49 ± 26.22	34.64 ± 19.47	2553.17 ± 232.07	0.22 ± 0.04	13.71 ± 4.05

### Calls

Urban and rural birds used the same types of calls, with no important differences detected in the relative percentages of each call type between urban and rural call repertoires. We detected no substantial differences in call duration or the peak rate of calls between urban and rural populations (Table 1). The only characteristic of calls that was affected by urban habitat was frequency range. Calls in urban areas had an average range of 2553.2 Hz compared with 2300.7 Hz in rural areas, an increase of 253 Hz (95% CI = 133.4, 373.0), or about 11%.

### Repertoire similarity

**Figure 3:**
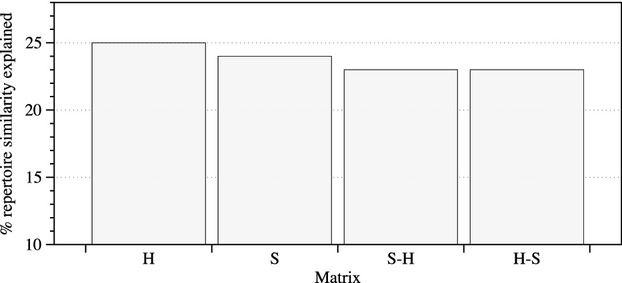
The percentage of variation between population syllable repertoires explained by each matrix as calculated by partial redundancy analysis. H, habitat matrix; S, spatial matrix; H–S, habitat matrix constrained by the spatial matrix; and S–H, the spatial matrix constrained by the habitat matrix.

Redundancy analyses indicated that 24% of the variance in population syllable repertoire was explained by geographic location, whereas habitat type explained 25% of the variance (Fig. 3). This indicates that both habitat and location were important predictors of repertoire similarity between populations. Specifically, latitude was the most important spatial variable, predicting 17% of the variation between dialects; noise, as the most important habitat variable, predicted 10% (Table 2).

**Table 2:** The contribution of variables in the habitat matrix (H) and spatial matrix (S) to the variation in the song syllable matrix explained by the RDA and partial RDA. H–S, habitat matrix, when spatial variation was removed (pure environmental variation); S–H, spatial matrix, when environmental variation was removed (pure spatial variation)

Variable	Matrix	Variation%	Matrix	Variation
Urban	H	9	H–S	9
Noise	H	10	H–S	9
Open	H	7	H–S	5
Y (latitude)	S	17	S–H	17
X (longitude)	S	7	S–H	6

## Discussion

Independent of geographic location, habitat type (urban vs. rural) was an important predictor of dialect similarity between populations. Although repertoires of urban and rural silvereye populations were similar in size, urban songs contained fewer syllables per song, and a higher percentage of trills within than rural songs. In addition, there was an increase in frequency range used for urban song syllables, and this increased range was also apparent in urban calls. These findings suggest that birds in urban environments may not only adaptively alter the spectral characteristics of their songs, but also preferentially use particular syllables or memes that are well suited to communication in the urban soundscape.

Partial redundancy analysis revealed that geographic location predicted 24% of the variance of syllable repertoire among populations, the majority of this variance being predicted by latitude. Environmental or habitat features predicted 25% of the variance, with noise being the strongest individual predictive variable. These results indicate that repertoire similarity (i.e., repertoires that contain more of the same syllable types in similar abundance) is influenced by both geographic location and habitat features. While the effect of geographic location is expected (due to factors such as isolation-by-distance and cultural drift (Irwin et al. 2008; Yoktan et al. 2011; Benedict and Bowie 2009), the strong influence of habitat (and especially noise) suggests possible convergence of song repertoire characteristics in urban habitats within the structure of these local dialects. Upon closer examination of the song and call repertoires in all locations, we can identify some of the characteristics that may be modified in urban environments, and how these changes fit with our predictions.

One previous study identified shifts in meme use in urban areas by comparing one urban and one rural population of birds (Cardoso and Atwell 2011b). By comparing multiple cities, we found that regardless of geographic location, urban silvereyes used a higher percentage of trills in their songs. This was contrary to our prediction that simpler or slower syllables might be used in urban areas for clarity, however may still support the acoustic adaptation hypothesis. These syllables may be important elements that punctuate songs, to attract or maintain the attention of receivers amidst noise. Alternatively, the increase in trill use may be related to other aspects of urban living. For instance, the lack of suitable habitat in urban areas may intensify competition for quality nesting space, and therefore heighten male–male competition, in turn affecting aspects of song (Hamao et al. 2011). Trills often have a performance limit, whereby low-quality singers are unable to quickly modulate syllables (Podos 1997; Bermudez-Cuamatzin et al. 2009), and therefore might have an important role in song contests between males. If male competition is intensified in urban parks, then the use of trills might become more important and feature more prominently in urban songs. Unfortunately density of males was not calculated in this study, but this would provide an interesting avenue for further research.

Another indication that urban habitat may be promoting convergent shifts in songs and calls was the finding that urban habitat predicted increased frequency ranges for both types of vocalizations; however, the effect contradicted our original prediction of narrower syllables in urban areas. Although there is evidence that urban birds decrease the overall bandwidth of their songs when raising the minimum frequency (Patricelli and Blickley 2006; Salaberria and Gil 2010), our study indicates that this may not always be the case, especially since the same populations of urban silvereyes are known to raise the minimum frequency of songs and calls (Potvin et al. 2011). Laboratory studies have demonstrated that broadband, frequency-modulated syllables are more difficult to distinguish from noise than pure tones (Lohr et al. 2003). However, the use of a larger range of frequencies both within syllables and within songs may be adaptive in an area where some frequencies are masked by urban noise.

Song syllables in urban areas were also sung at a slightly slower tempo than rural syllables, as predicted, providing further evidence for an adaptive urban effect on the structure of song. Peak rate or tempo can be considered an important measure of syllable complexity, analogous to trill rate in other species, as it involves repeated wide frequency modulations (Catchpole and Slater 2008). It is possible that urban birds are adjusting the peak rate of their syllables to increase syllable information transmission in noisy environments, similar to the adjustment of syllable rate within songs (Bermudez-Cuamatzin et al. 2011). This decrease in modulation speed may lower the performance limit of frequency modulation and enable urban individuals to increase the frequency range of such syllables. This would imply that such syllables might retain their potential function as quality indicators, while also transmitting more effectively in urban areas. Alternatively, slower syllables may be more suited to urban environments due to the effects of urban architecture. There is evidence that elements of the urban acoustic environment other than noise (e.g., surrounding structures) may select for slower vocalization types, similar to phenomena found in natural environments (Mockford et al. 2011). This may also be why noise levels alone do not entirely explain changes in urban song characteristics (Warren et al. 2006; Verzijden et al. 2010; Mockford et al. 2011; Potvin et al. 2011).

Urban songs contained fewer syllables per song than those sung in rural areas. This is consistent with previous findings that urban silvereye songs contain fewer syllables per second, even though the duration of urban songs is similar to that of rural songs (Potvin et al. 2011). However, both urban and rural populations had similar repertoire sizes. Thus, if individual repertoire size is an important feature of silvereye song, urban silvereyes would need to sing more songs to reveal their full syllable repertoire. Singing at a slower rate in urban areas could improve the clarity of songs against surrounding background noise (Nemeth and Brumm 2010; Mockford et al. 2011), but such an adaptation may be costly if urban birds need to spend more time singing at dawn (postponing morning feeding) to include their entire repertoire. Future studies focusing on the importance of repertoire size and length of dawn chorus in different habitats in this species are required to clearly understand the benefits and costs of such behavioral changes.

We found that certain features of songs and calls are consistently altered in urban environments, probably to improve signal transmission or detectability, and that these adjustments are present regardless of the geographic dialect of the population. Increasing minimum frequency, slowing syllable rate, slowing syllable peak rate, and including a higher percentage of trills in songs may all aid in the transmission, detectability, and/or discrimination of signals in urban habitats, as evidenced by the consistent changes in multiple city populations over large geographic areas. However, if calls need to retain an individual signature, it follows that there are fewer options for improving transmission of individually distinct calls than songs. The former may be constrained to frequency and amplitude shifts; by contrast, urban song syllables may be altered within the context of a geographic repertoire. Our findings demonstrate that although the geographic position of a population is an important determinant of its song repertoire, urban populations (regardless of geographic location) are consistently shaping these repertoires in adaptive ways that are likely to increase transmission in urban noise, providing further support for the acoustic adaptation hypothesis in an urban context.

## Supporting Information

**Appendix S1.** Short call (a), variable call (b), and linear call (c) of two different silvereye individuals. Number of peaks, length of call, and overall shape determined the category of call, which was stereotypical. A fourth call, the “chip” call, constituted of one simple, short (1 ms), unwavering tone. Peaks were defined as the highest points above the average frequency, used as an objective measure of frequency modulation and therefore complexity of a syllable (peaks/sec). In this figure, the short call of individual one has two peaks, the variable call has four peaks, and the linear call has one peak.

**Appendix S2.** Map of the seven geographic locations sampled in Eastern Australia. Sampling was completed at two sites – one urban and one rural – at each geographic location, for a total of 14 sites. Sample sizes indicate number of individuals for which dawn chorus and contact calls were recorded at each urban and rural sites, respectively.
