# Supplementary figures and images for: Song convergence in multiple urban populations of silvereyes (Zosterops lateralis)
Source: Ecol Evol. 2012 Jul 16;2(8):1977–84. doi: 10.1002/ece3.320 (PMC3434000; doi:10.1002/ece3.320)

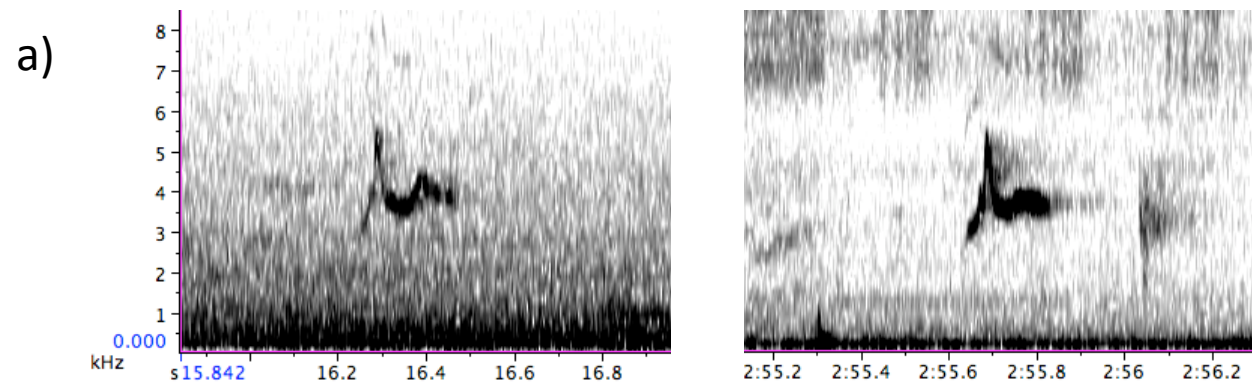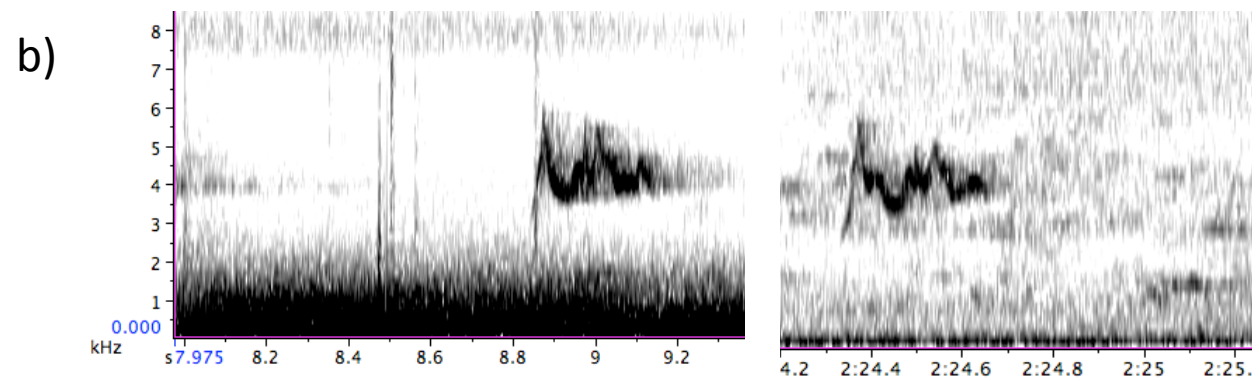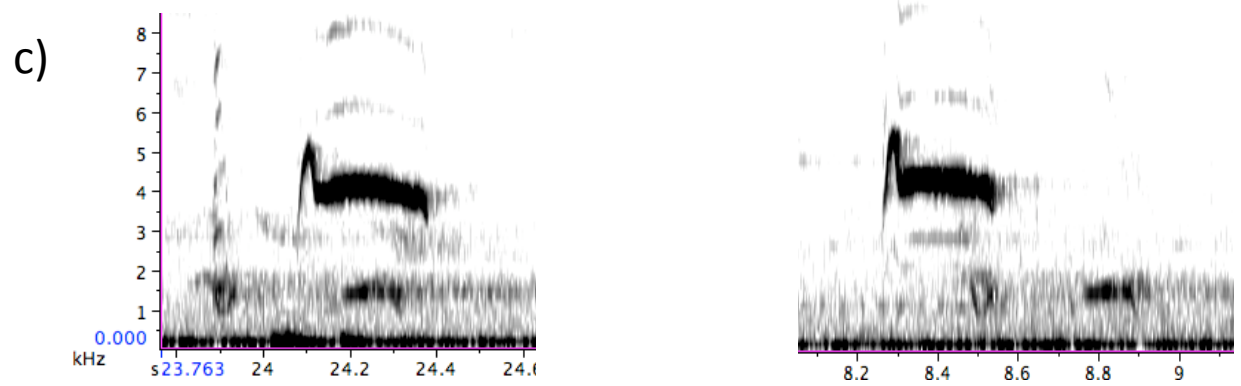

Supplement: Supplementary file 1 [file ece30002-1977-SD1.pdf]

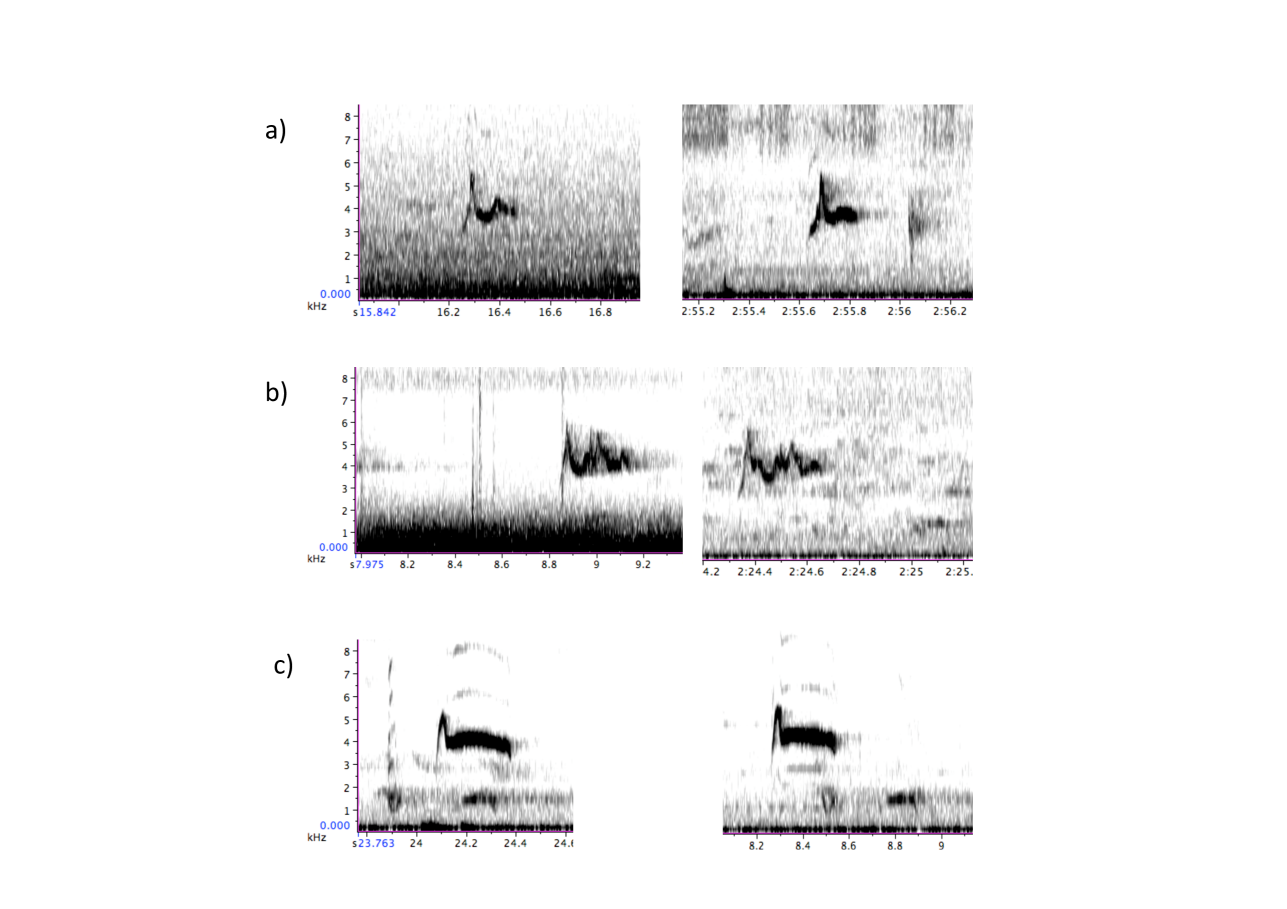

Supplement: Supplementary file 2 [file ece30002-1977-SD3.png]

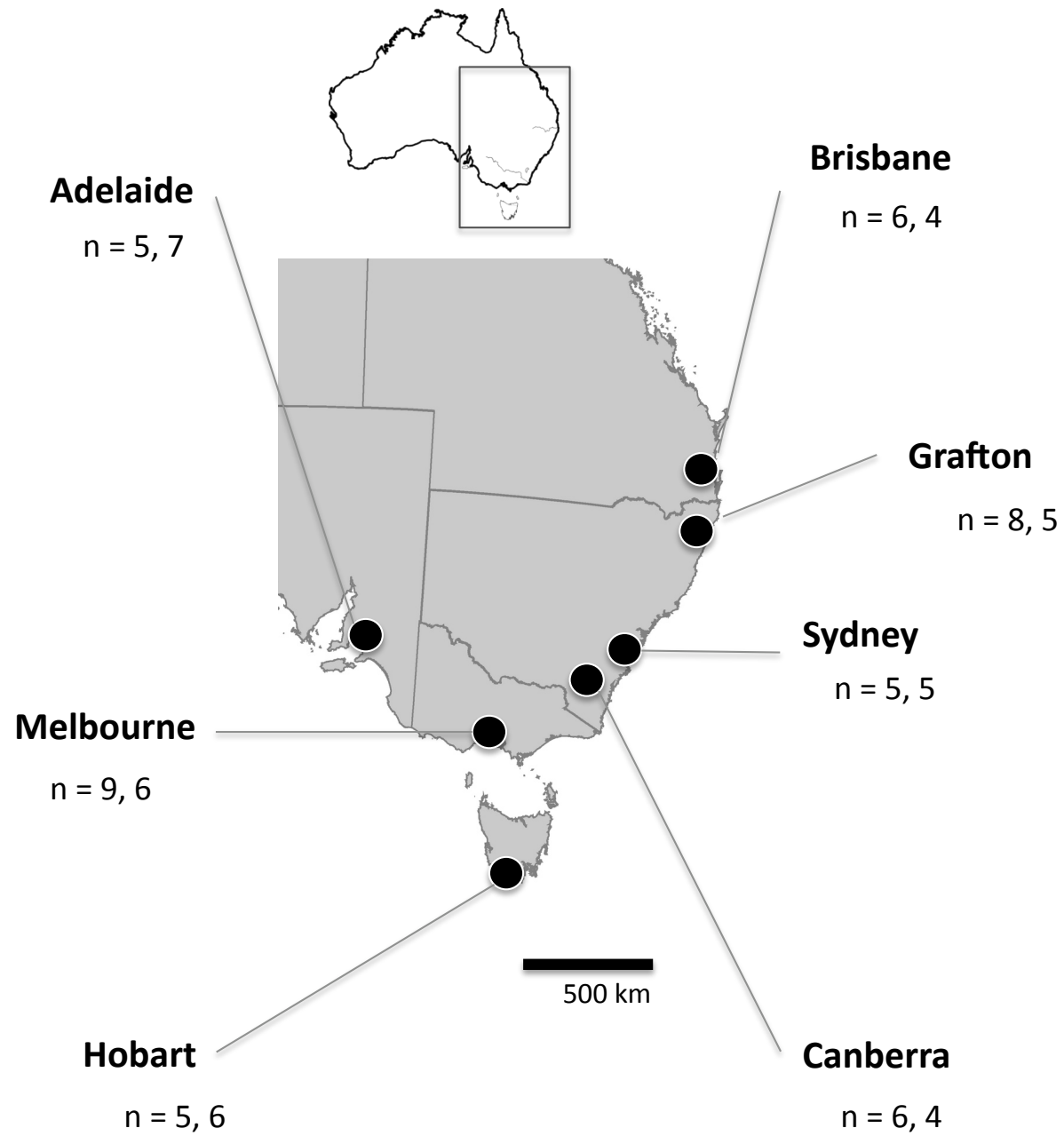

Supplement: Supplementary file 4 [file ece30002-1977-SD2.pdf]

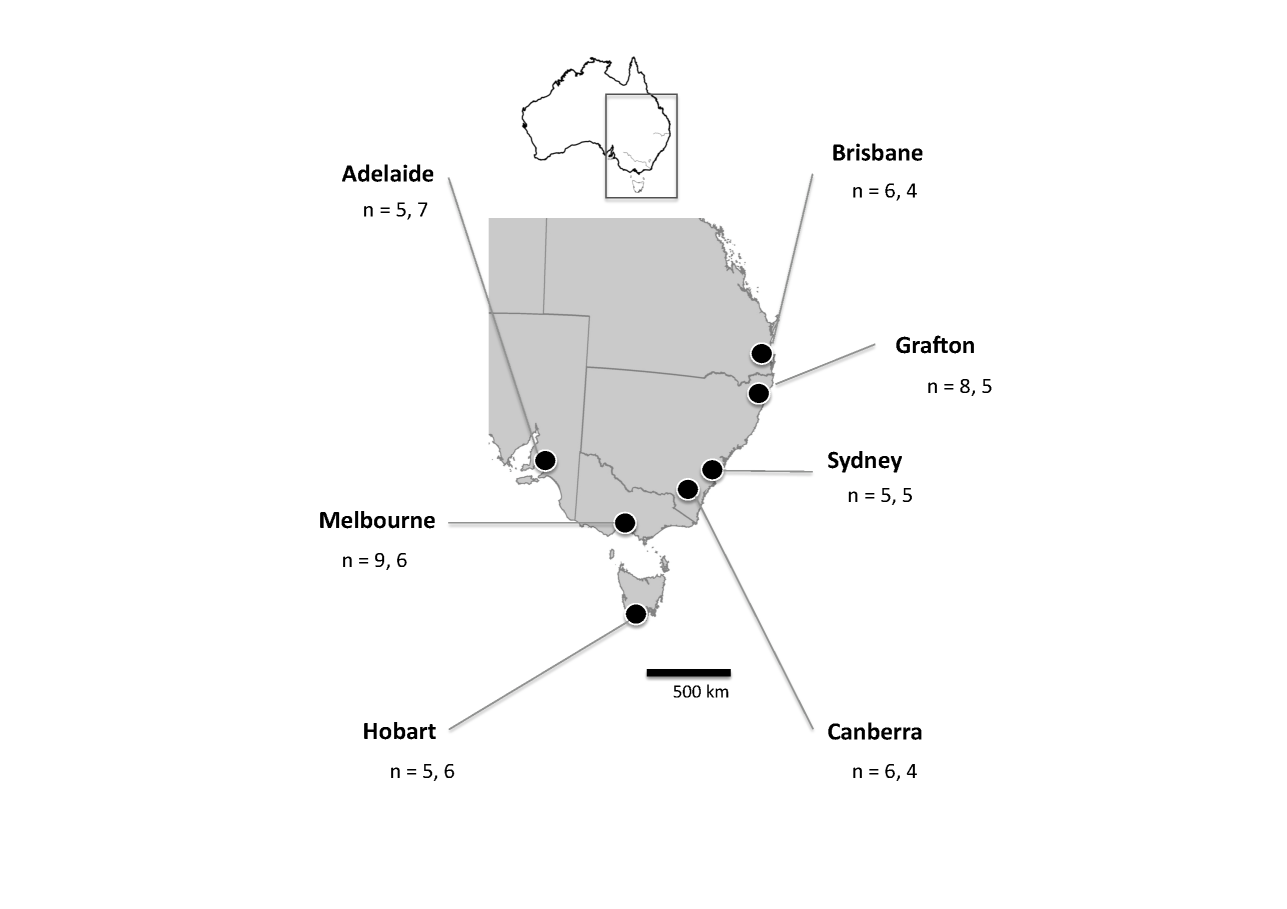

Supplement: Supplementary file 5 [file ece30002-1977-SD4.png]
